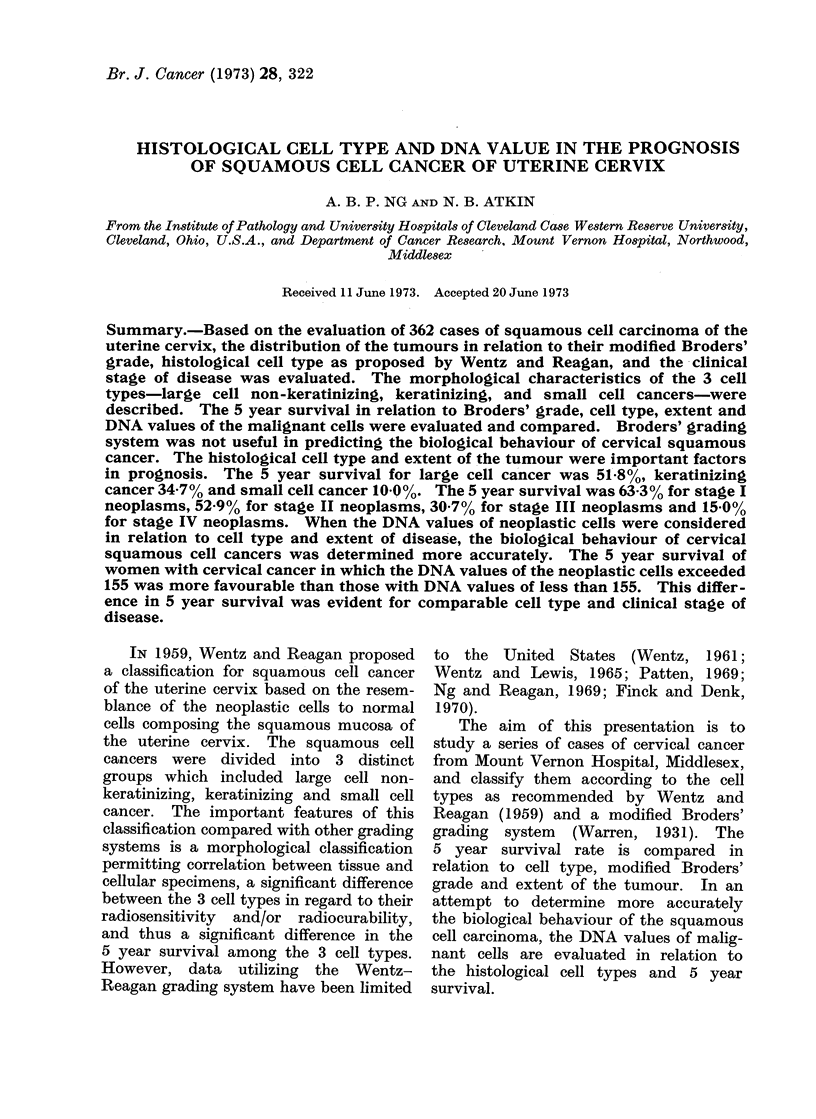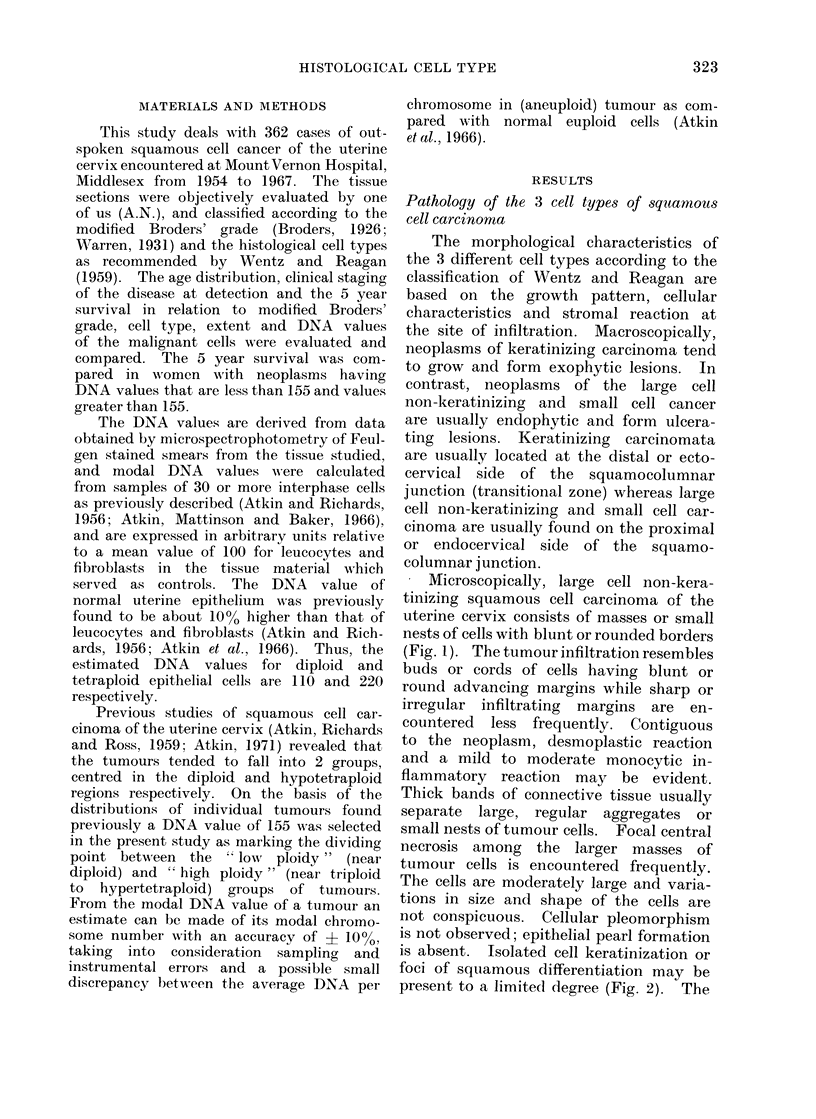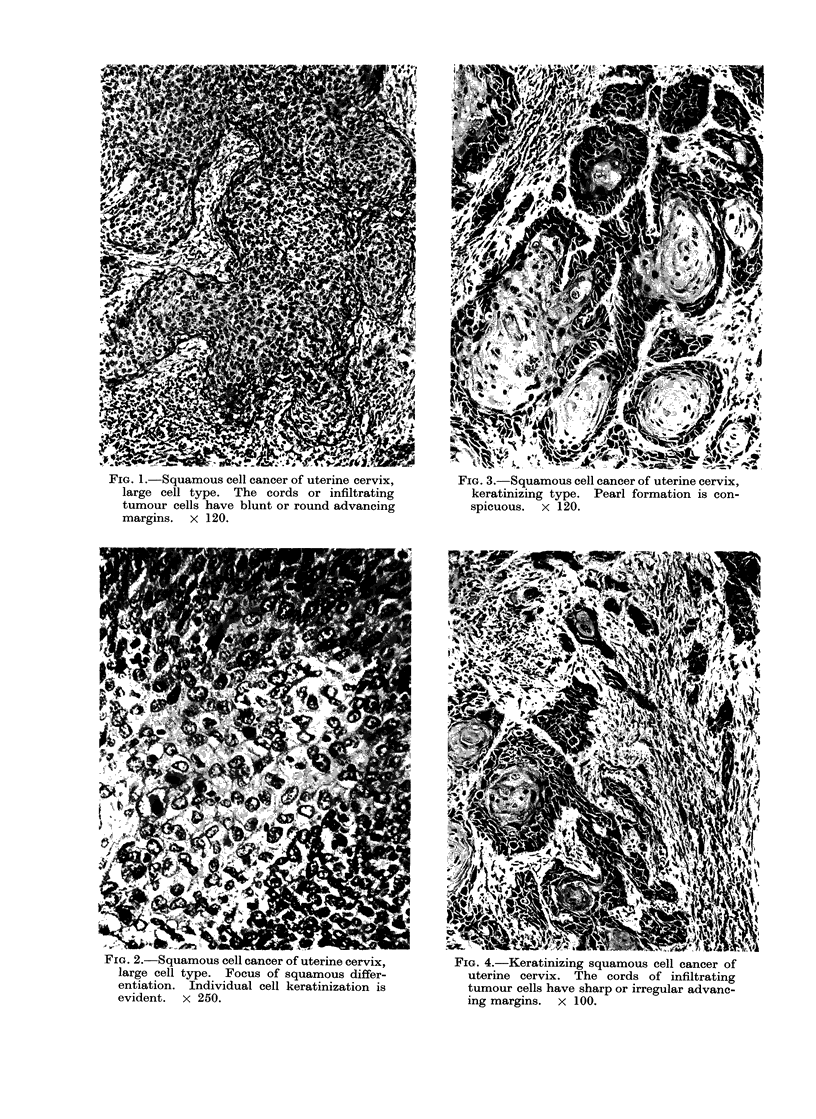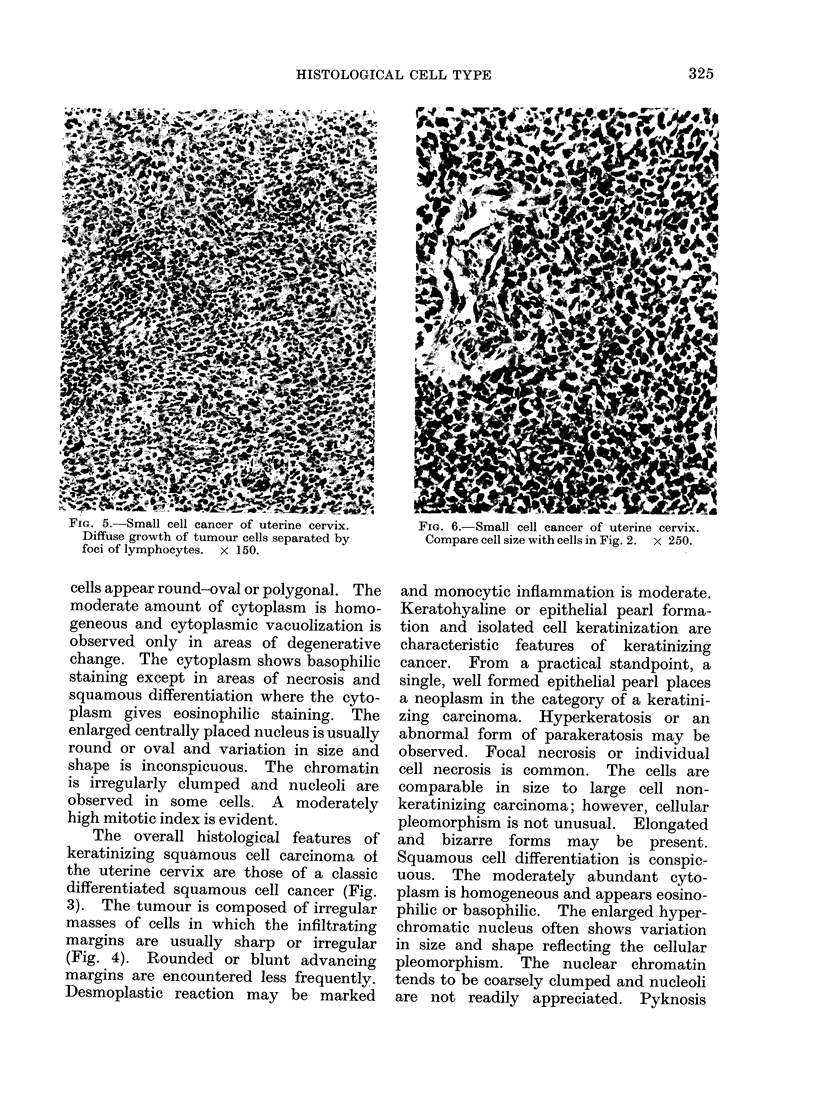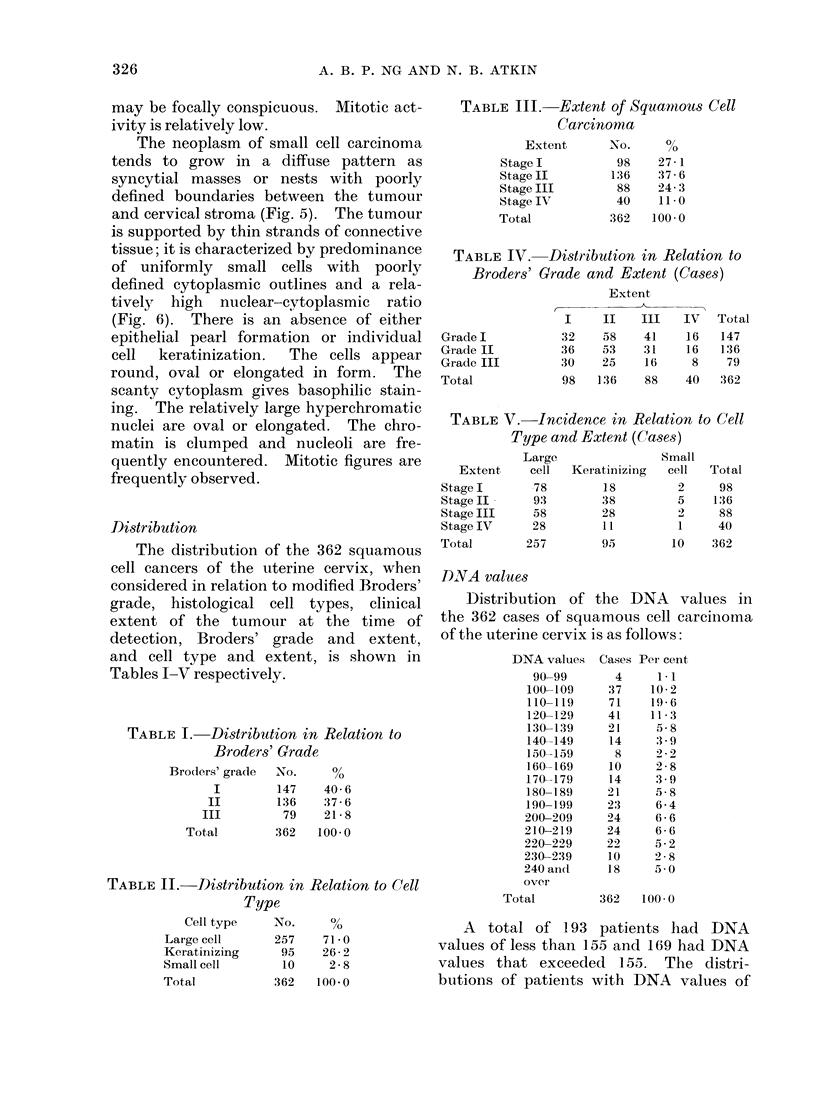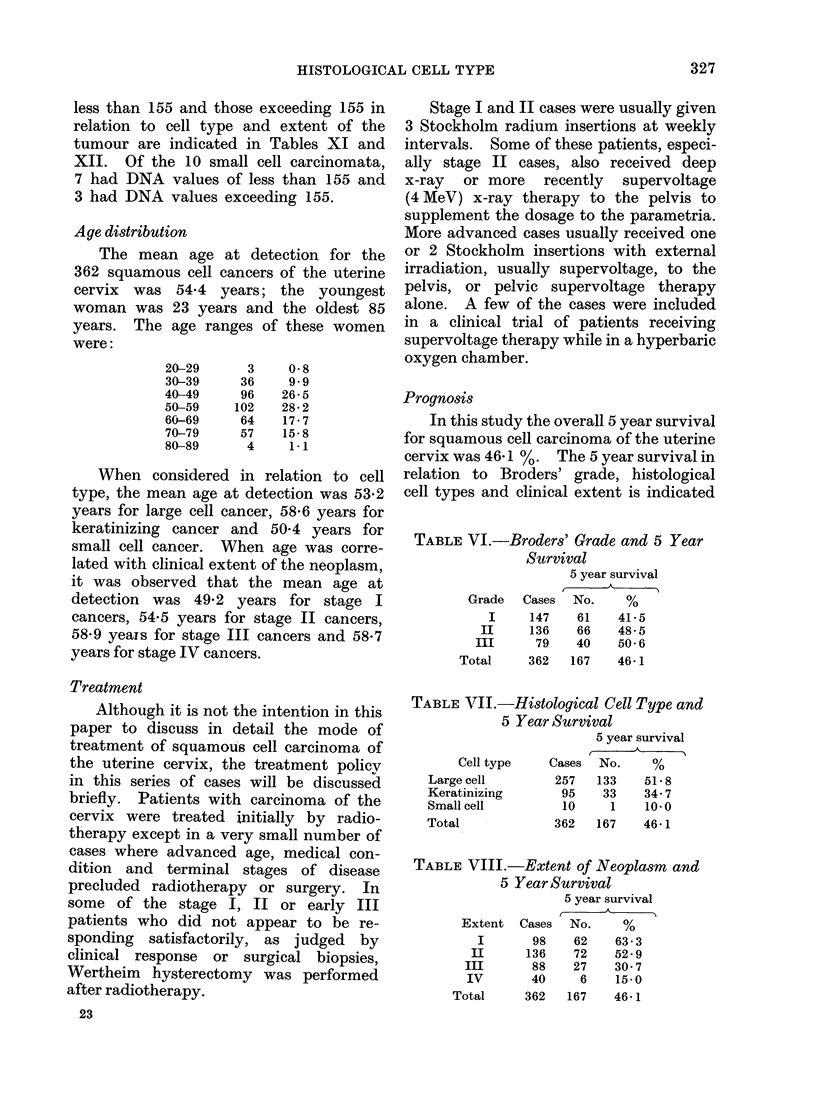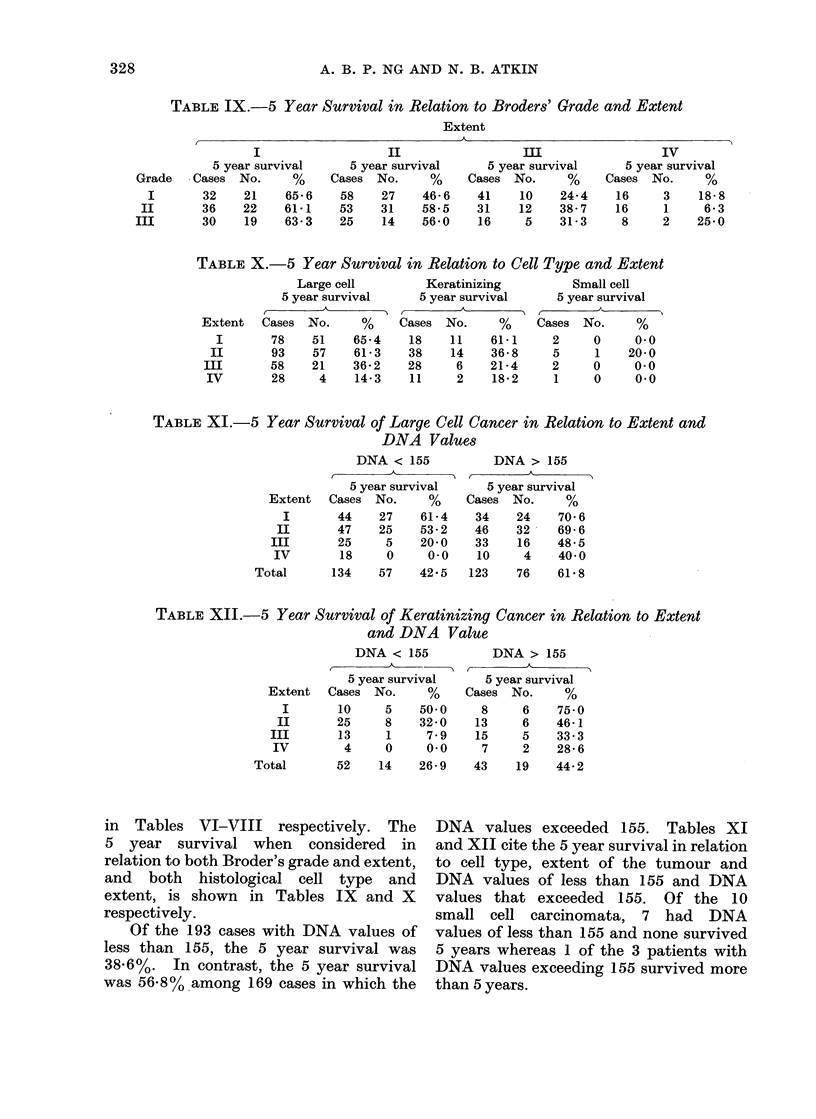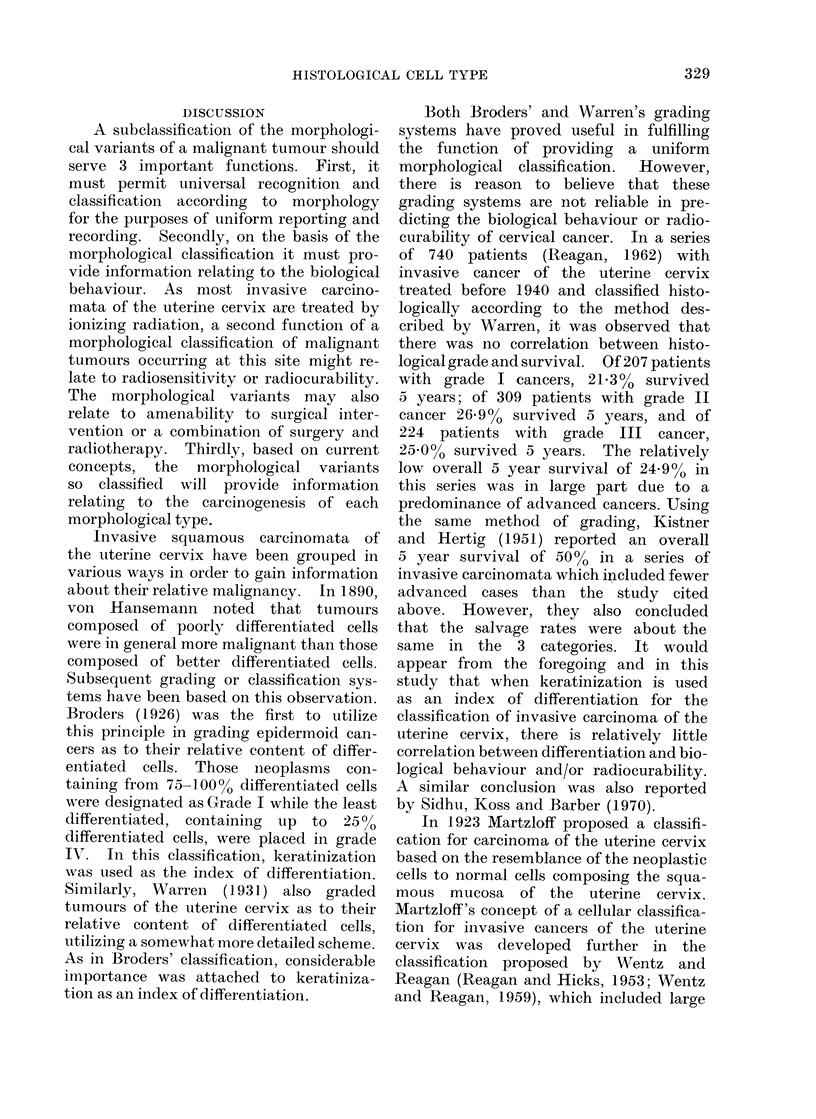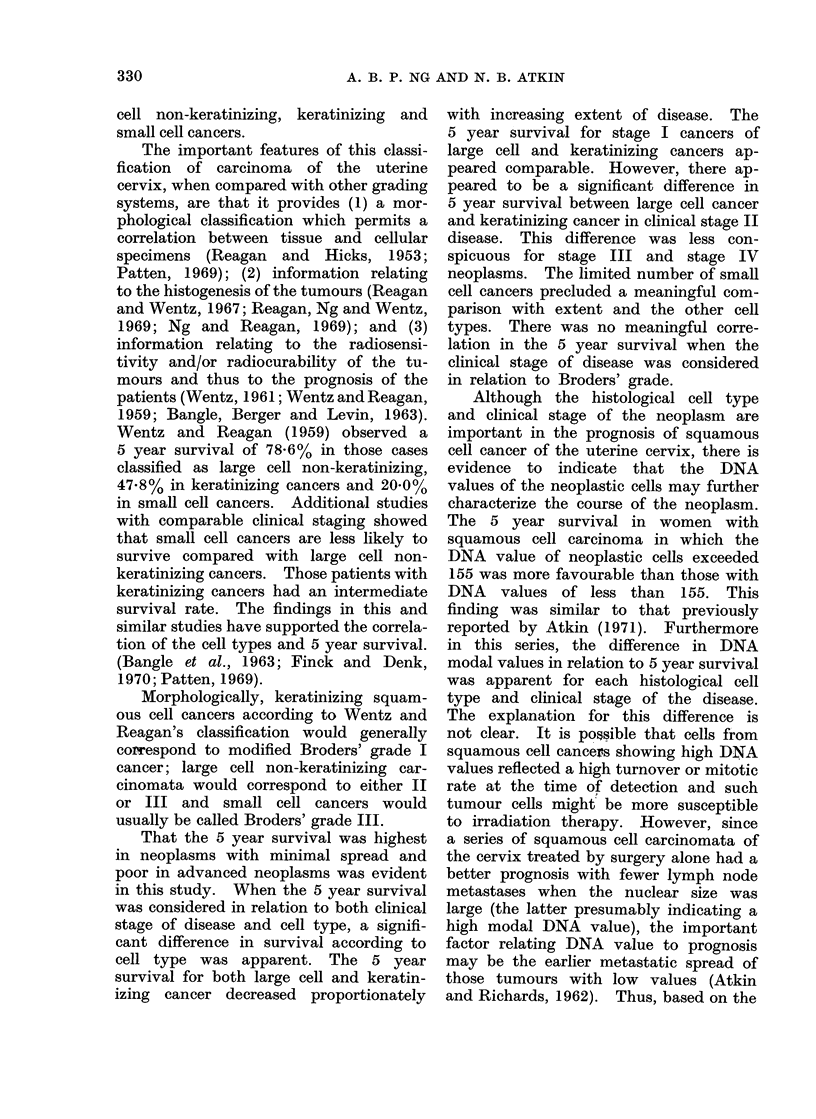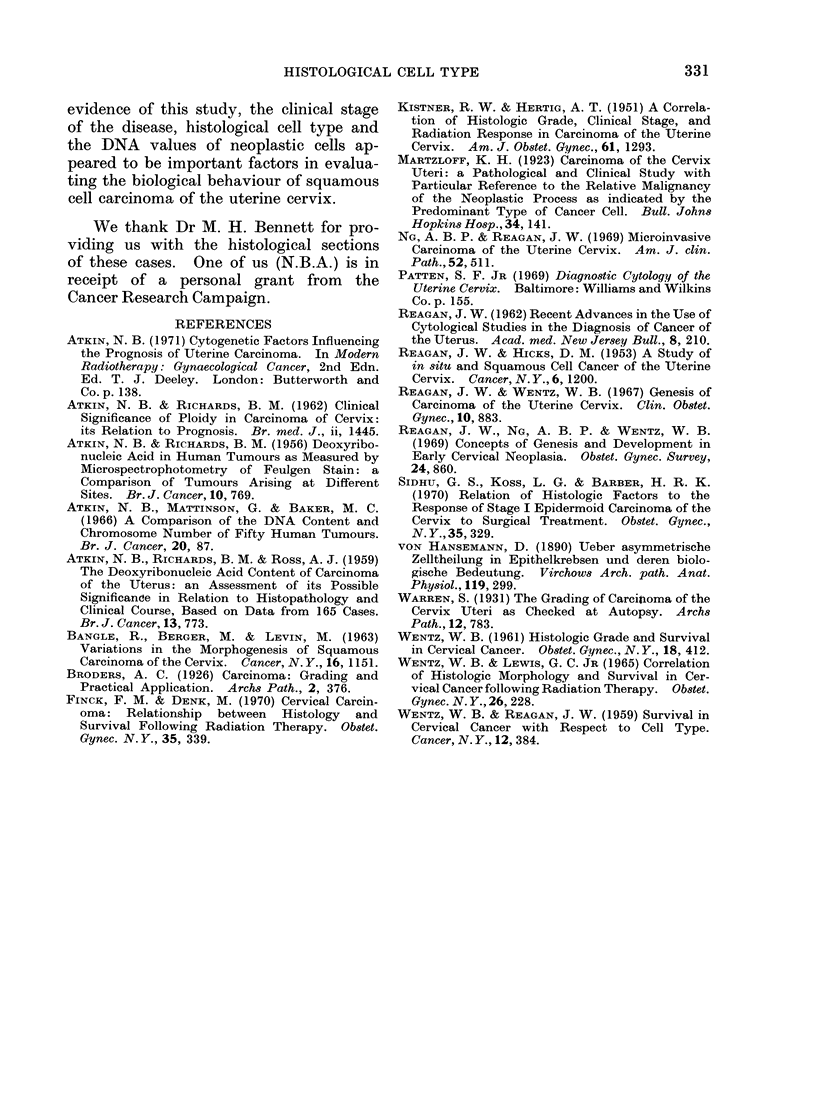# Histological Cell Type and DNA Value in the Prognosis of Squamous Cell Cancer of Uterine Cervix

**DOI:** 10.1038/bjc.1973.155

**Published:** 1973-10

**Authors:** A. B. P. Ng, N. B. Atkin

## Abstract

**Images:**


*Br. J. Cancer* (1973) **28**, 322

### HISTOLOGICAL CELL TYPE AND DNA VALUE IN THE PROGNOSIS OF SQUAMOUS CELL CANCER OF UTERINE CERVIX

A. B. P. NG AND N. B. ATKIN

*From the Institute of Pathology and University Hospitals of Cleveland Case Western Reserve University, Cleveland, Ohio, U.S.A., and Department of Cancer Research, Mount Vernon Hospital, Northwood, Middlesex*

Received 11 June 1973. Accepted 20 June 1973

**Summary.—Based on the evaluation of 362 cases of squamous cell carcinoma of the uterine cervix, the distribution of the tumours in relation to their modified Broders' grade, histological cell type as proposed by Wentz and Reagan, and the clinical stage of disease was evaluated. The morphological characteristics of the 3 cell types—large cell non-keratinizing, keratinizing, and small cell cancers—were described. The 5 year survival in relation to Broders' grade, cell type, extent and DNA values of the malignant cells were evaluated and compared. Broders' grading system was not useful in predicting the biological behaviour of cervical squamous cancer. The histological cell type and extent of the tumour were important factors in prognosis. The 5 year survival for large cell cancer was 51·8%, keratinizing cancer 34·7% and small cell cancer 10·0%. The 5 year survival was 63·3% for stage I neoplasms, 52·9% for stage II neoplasms, 30·7% for stage III neoplasms and 15·0% for stage IV neoplasms. When the DNA values of neoplastic cells were considered in relation to cell type and extent of disease, the biological behaviour of cervical squamous cell cancers was determined more accurately. The 5 year survival of women with cervical cancer in which the DNA values of the neoplastic cells exceeded 155 was more favourable than those with DNA values of less than 155. This difference in 5 year survival was evident for comparable cell type and clinical stage of disease.**

In 1959, Wentz and Reagan proposed a classification for squamous cell cancer of the uterine cervix based on the resemblance of the neoplastic cells to normal cells composing the squamous mucosa of the uterine cervix. The squamous cell cancers were divided into 3 distinct groups which included large cell non-keratinizing, keratinizing and small cell cancer. The important features of this classification compared with other grading systems is a morphological classification permitting correlation between tissue and cellular specimens, a significant difference between the 3 cell types in regard to their radiosensitivity and/or radiocurability, and thus a significant difference in the 5 year survival among the 3 cell types. However, data utilizing the Wentz–Reagan grading system have been limited to the United States (Wentz, 1961; Wentz and Lewis, 1965; Patten, 1969; Ng and Reagan, 1969; Finck and Denk, 1970).

The aim of this presentation is to study a series of cases of cervical cancer from Mount Vernon Hospital, Middlesex, and classify them according to the cell types as recommended by Wentz and Reagan (1959) and a modified Broders' grading system (Warren, 1931). The 5 year survival rate is compared in relation to cell type, modified Broders' grade and extent of the tumour. In an attempt to determine more accurately the biological behaviour of the squamous cell carcinoma, the DNA values of malignant cells are evaluated in relation to the histological cell types and 5 year survival.

### MATERIALS AND METHODS

This study deals with 362 cases of outspoken squamous cell cancer of the uterine cervix encountered at Mount Vernon Hospital, Middlesex from 1954 to 1967. The tissue sections were objectively evaluated by one of us (A.N.), and classified according to the modified Broders' grade (Broders, 1926; Warren, 1931) and the histological cell types as recommended by Wentz and Reagan (1959). The age distribution, clinical staging of the disease at detection and the 5 year survival in relation to modified Broders' grade, cell type, extent and DNA values of the malignant cells were evaluated and compared. The 5 year survival was compared in women with neoplasms having DNA values that are less than 155 and values greater than 155.

The DNA values are derived from data obtained by microspectrophotometry of Feulgen stained smears from the tissue studied, and modal DNA values were calculated from samples of 30 or more interphase cells as previously described (Atkin and Richards, 1956; Atkin, Mattinson and Baker, 1966), and are expressed in arbitrary units relative to a mean value of 100 for leucocytes and fibroblasts in the tissue material which served as controls. The DNA value of normal uterine epithelium was previously found to be about 10% higher than that of leucocytes and fibroblasts (Atkin and Richards, 1956; Atkin *et al.*, 1966). Thus, the estimated DNA values for diploid and tetraploid epithelial cells are 110 and 220 respectively.

Previous studies of squamous cell carcinoma of the uterine cervix (Atkin, Richards and Ross, 1959; Atkin, 1971) revealed that the tumours tended to fall into 2 groups, centred in the diploid and hypotetraploid regions respectively. On the basis of the distributions of individual tumours found previously a DNA value of 155 was selected in the present study as marking the dividing point between the "low ploidy" (near diploid) and "high ploidy" (near triploid to hypertetraploid) groups of tumours. From the modal DNA value of a tumour an estimate can be made of its modal chromosome number with an accuracy of $\pm$ 10%, taking into consideration sampling and instrumental errors and a possible small discrepancy between the average DNA per chromosome in (aneuploid) tumour as compared with normal euploid cells (Atkin *et al.*, 1966).

### RESULTS

### *Pathology of the 3 cell types of squamous cell carcinoma*

The morphological characteristics of the 3 different cell types according to the classification of Wentz and Reagan are based on the growth pattern, cellular characteristics and stromal reaction at the site of infiltration. Macroscopically, neoplasms of keratinizing carcinoma tend to grow and form exophytic lesions. In contrast, neoplasms of the large cell non-keratinizing and small cell cancer are usually endophytic and form ulcerating lesions. Keratinizing carcinomata are usually located at the distal or ectocervical side of the squamocolumnar junction (transitional zone) whereas large cell non-keratinizing and small cell carcinoma are usually found on the proximal or endocervical side of the squamocolumnar junction.

Microscopically, large cell non-keratinizing squamous cell carcinoma of the uterine cervix consists of masses or small nests of cells with blunt or rounded borders (Fig. 1). The tumour infiltration resembles buds or cords of cells having blunt or round advancing margins while sharp or irregular infiltrating margins are encountered less frequently. Contiguous to the neoplasm, desmoplastic reaction and a mild to moderate monocytic inflammatory reaction may be evident. Thick bands of connective tissue usually separate large, regular aggregates or small nests of tumour cells. Focal central necrosis among the larger masses of tumour cells is encountered frequently. The cells are moderately large and variations in size and shape of the cells are not conspicuous. Cellular pleomorphism is not observed; epithelial pearl formation is absent. Isolated cell keratinization or foci of squamous differentiation may be present to a limited degree (Fig. 2). The

Fig. 1.—Squamous cell cancer of uterine cervix, large cell type. The cords or infiltrating tumour cells have blunt or round advancing margins. × 120.

Fig. 3.—Squamous cell cancer of uterine cervix, keratinizing type. Pearl formation is conspicuous. × 120.

Fig. 2.—Squamous cell cancer of uterine cervix, large cell type. Focus of squamous differentiation. Individual cell keratinization is evident. × 250.

Fig. 4.—Keratinizing squamous cell cancer of uterine cervix. The cords of infiltrating tumour cells have sharp or irregular advancing margins. × 100.

FIG. 5.—Small cell cancer of uterine cervix. Diffuse growth of tumour cells separated by foci of lymphocytes. × 150.

FIG. 6.—Small cell cancer of uterine cervix. Compare cell size with cells in Fig. 2. × 250.

cells appear round–oval or polygonal. The moderate amount of cytoplasm is homogeneous and cytoplasmic vacuolization is observed only in areas of degenerative change. The cytoplasm shows basophilic staining except in areas of necrosis and squamous differentiation where the cytoplasm gives eosinophilic staining. The enlarged centrally placed nucleus is usually round or oval and variation in size and shape is inconspicuous. The chromatin is irregularly clumped and nucleoli are observed in some cells. A moderately high mitotic index is evident.

The overall histological features of keratinizing squamous cell carcinoma of the uterine cervix are those of a classic differentiated squamous cell cancer (Fig. 3). The tumour is composed of irregular masses of cells in which the infiltrating margins are usually sharp or irregular (Fig. 4). Rounded or blunt advancing margins are encountered less frequently. Desmoplastic reaction may be marked and monocytic inflammation is moderate. Keratohyaline or epithelial pearl formation and isolated cell keratinization are characteristic features of keratinizing cancer. From a practical standpoint, a single, well formed epithelial pearl places a neoplasm in the category of a keratinizing carcinoma. Hyperkeratosis or an abnormal form of parakeratosis may be observed. Focal necrosis or individual cell necrosis is common. The cells are comparable in size to large cell non-keratinizing carcinoma; however, cellular pleomorphism is not unusual. Elongated and bizarre forms may be present. Squamous cell differentiation is conspicuous. The moderately abundant cytoplasm is homogeneous and appears eosinophilic or basophilic. The enlarged hyperchromatic nucleus often shows variation in size and shape reflecting the cellular pleomorphism. The nuclear chromatin tends to be coarsely clumped and nucleoli are not readily appreciated. Pyknosis may be focally conspicuous. Mitotic activity is relatively low.

The neoplasm of small cell carcinoma tends to grow in a diffuse pattern as syncytial masses or nests with poorly defined boundaries between the tumour and cervical stroma (Fig. 5). The tumour is supported by thin strands of connective tissue; it is characterized by predominance of uniformly small cells with poorly defined cytoplasmic outlines and a relatively high nuclear–cytoplasmic ratio (Fig. 6). There is an absence of either epithelial pearl formation or individual cell keratinization. The cells appear round, oval or elongated in form. The scanty cytoplasm gives basophilic staining. The relatively large hyperchromatic nuclei are oval or elongated. The chromatin is clumped and nucleoli are frequently encountered. Mitotic figures are frequently observed.

### *Distribution*

The distribution of the 362 squamous cell cancers of the uterine cervix, when considered in relation to modified Broders' grade, histological cell types, clinical extent of the tumour at the time of detection, Broders' grade and extent, and cell type and extent, is shown in Tables I–V respectively.

TABLE I.—*Distribution in Relation to Broders' Grade*

| Broders' grade | No. | % |
|---|---|---|
| I | 147 | 40·6 |
| II | 136 | 37·6 |
| III | 79 | 21·8 |
| Total | 362 | 100·0 |

TABLE II.—*Distribution in Relation to Cell Type*

| Cell type | No. | % |
|---|---|---|
| Large cell | 257 | 71·0 |
| Keratinizing | 95 | 26·2 |
| Small cell | 10 | 2·8 |
| Total | 362 | 100·0 |

TABLE III.—*Extent of Squamous Cell Carcinoma*

| Extent | No. | % |
|---|---|---|
| Stage I | 98 | 27·1 |
| Stage II | 136 | 37·6 |
| Stage III | 88 | 24·3 |
| Stage IV | 40 | 11·0 |
| Total | 362 | 100·0 |

TABLE IV.—*Distribution in Relation to Broders' Grade and Extent (Cases)*

| | Extent | | | | |
|---|---|---|---|---|---|
| | I | II | III | IV | Total |
| Grade I | 32 | 58 | 41 | 16 | 147 |
| Grade II | 36 | 53 | 31 | 16 | 136 |
| Grade III | 30 | 25 | 16 | 8 | 79 |
| Total | 98 | 136 | 88 | 40 | 362 |

TABLE V.—*Incidence in Relation to Cell Type and Extent (Cases)*

| Extent | Large cell | Keratinizing | Small cell | Total |
|---|---|---|---|---|
| Stage I | 78 | 18 | 2 | 98 |
| Stage II | 93 | 38 | 5 | 136 |
| Stage III | 58 | 28 | 2 | 88 |
| Stage IV | 28 | 11 | 1 | 40 |
| Total | 257 | 95 | 10 | 362 |

### *DNA values*

Distribution of the DNA values in the 362 cases of squamous cell carcinoma of the uterine cervix is as follows:

| DNA values | Cases | Per cent |
|---|---|---|
| 90–99 | 4 | 1·1 |
| 100–109 | 37 | 10·2 |
| 110–119 | 71 | 19·6 |
| 120–129 | 41 | 11·3 |
| 130–139 | 21 | 5·8 |
| 140–149 | 14 | 3·9 |
| 150–159 | 8 | 2·2 |
| 160–169 | 10 | 2·8 |
| 170–179 | 14 | 3·9 |
| 180–189 | 21 | 5·8 |
| 190–199 | 23 | 6·4 |
| 200–209 | 24 | 6·6 |
| 210–219 | 24 | 6·6 |
| 220–229 | 22 | 5·2 |
| 230–239 | 10 | 2·8 |
| 240 and over | 18 | 5·0 |
| Total | 362 | 100·0 |

A total of 193 patients had DNA values of less than 155 and 169 had DNA values that exceeded 155. The distributions of patients with DNA values of less than 155 and those exceeding 155 in relation to cell type and extent of the tumour are indicated in Tables XI and XII. Of the 10 small cell carcinomata, 7 had DNA values of less than 155 and 3 had DNA values exceeding 155.

*Age distribution*

The mean age at detection for the 362 squamous cell cancers of the uterine cervix was 54·4 years; the youngest woman was 23 years and the oldest 85 years. The age ranges of these women were:

| | | |
|---|---|---|
| 20–29 | 3 | 0·8 |
| 30–39 | 36 | 9·9 |
| 40–49 | 96 | 26·5 |
| 50–59 | 102 | 28·2 |
| 60–69 | 64 | 17·7 |
| 70–79 | 57 | 15·8 |
| 80–89 | 4 | 1·1 |

When considered in relation to cell type, the mean age at detection was 53·2 years for large cell cancer, 58·6 years for keratinizing cancer and 50·4 years for small cell cancer. When age was correlated with clinical extent of the neoplasm, it was observed that the mean age at detection was 49·2 years for stage I cancers, 54·5 years for stage II cancers, 58·9 yeaιs for stage III cancers and 58·7 years for stage IV cancers.

*Treatment*

Although it is not the intention in this paper to discuss in detail the mode of treatment of squamous cell carcinoma of the uterine cervix, the treatment policy in this series of cases will be discussed briefly. Patients with carcinoma of the cervix were treated initially by radiotherapy except in a very small number of cases where advanced age, medical condition and terminal stages of disease precluded radiotherapy or surgery. In some of the stage I, II or early III patients who did not appear to be responding satisfactorily, as judged by clinical response or surgical biopsies, Wertheim hysterectomy was performed after radiotherapy.

Stage I and II cases were usually given 3 Stockholm radium insertions at weekly intervals. Some of these patients, especially stage II cases, also received deep x-ray or more recently supervoltage (4 MeV) x-ray therapy to the pelvis to supplement the dosage to the parametria. More advanced cases usually received one or 2 Stockholm insertions with external irradiation, usually supervoltage, to the pelvis, or pelvic supervoltage therapy alone. A few of the cases were included in a clinical trial of patients receiving supervoltage therapy while in a hyperbaric oxygen chamber.

*Prognosis*

In this study the overall 5 year survival for squamous cell carcinoma of the uterine cervix was 46·1 %. The 5 year survival in relation to Broders' grade, histological cell types and clinical extent is indicated

TABLE VI.—*Broders' Grade and 5 Year Survival*

| Grade | Cases | 5 year survival No. | % |
|---|---|---|---|
| I | 147 | 61 | 41·5 |
| II | 136 | 66 | 48·5 |
| III | 79 | 40 | 50·6 |
| Total | 362 | 167 | 46·1 |

TABLE VII.—*Histological Cell Type and 5 Year Survival*

| Cell type | Cases | 5 year survival No. | % |
|---|---|---|---|
| Large cell | 257 | 133 | 51·8 |
| Keratinizing | 95 | 33 | 34·7 |
| Small cell | 10 | 1 | 10·0 |
| Total | 362 | 167 | 46·1 |

TABLE VIII.—*Extent of Neoplasm and 5 Year Survival*

| Extent | Cases | 5 year survival No. | % |
|---|---|---|---|
| I | 98 | 62 | 63·3 |
| II | 136 | 72 | 52·9 |
| III | 88 | 27 | 30·7 |
| IV | 40 | 6 | 15·0 |
| Total | 362 | 167 | 46·1 |

TABLE IX.—*5 Year Survival in Relation to Broders' Grade and Extent*

| | Extent | | | | | | | | | | | |
|---|---|---|---|---|---|---|---|---|---|---|---|---|
| | I | | | II | | | III | | | IV | | |
| | 5 year survival | | | 5 year survival | | | 5 year survival | | | 5 year survival | | |
| Grade | Cases | No. | % | Cases | No. | % | Cases | No. | % | Cases | No. | % |
| I | 32 | 21 | 65·6 | 58 | 27 | 46·6 | 41 | 10 | 24·4 | 16 | 3 | 18·8 |
| II | 36 | 22 | 61·1 | 53 | 31 | 58·5 | 31 | 12 | 38·7 | 16 | 1 | 6·3 |
| III | 30 | 19 | 63·3 | 25 | 14 | 56·0 | 16 | 5 | 31·3 | 8 | 2 | 25·0 |

TABLE X.—*5 Year Survival in Relation to Cell Type and Extent*

| | Large cell 5 year survival | | | Keratinizing 5 year survival | | | Small cell 5 year survival | | |
|---|---|---|---|---|---|---|---|---|---|
| Extent | Cases | No. | % | Cases | No. | % | Cases | No. | % |
| I | 78 | 51 | 65·4 | 18 | 11 | 61·1 | 2 | 0 | 0·0 |
| II | 93 | 57 | 61·3 | 38 | 14 | 36·8 | 5 | 1 | 20·0 |
| III | 58 | 21 | 36·2 | 28 | 6 | 21·4 | 2 | 0 | 0·0 |
| IV | 28 | 4 | 14·3 | 11 | 2 | 18·2 | 1 | 0 | 0·0 |

TABLE XI.—*5 Year Survival of Large Cell Cancer in Relation to Extent and DNA Values*

| | DNA < 155 | | | DNA > 155 | | |
|---|---|---|---|---|---|---|
| | 5 year survival | | | 5 year survival | | |
| Extent | Cases | No. | % | Cases | No. | % |
| I | 44 | 27 | 61·4 | 34 | 24 | 70·6 |
| II | 47 | 25 | 53·2 | 46 | 32 | 69·6 |
| III | 25 | 5 | 20·0 | 33 | 16 | 48·5 |
| IV | 18 | 0 | 0·0 | 10 | 4 | 40·0 |
| Total | 134 | 57 | 42·5 | 123 | 76 | 61·8 |

TABLE XII.—*5 Year Survival of Keratinizing Cancer in Relation to Extent and DNA Value*

| | DNA < 155 | | | DNA > 155 | | |
|---|---|---|---|---|---|---|
| | 5 year survival | | | 5 year survival | | |
| Extent | Cases | No. | % | Cases | No. | % |
| I | 10 | 5 | 50·0 | 8 | 6 | 75·0 |
| II | 25 | 8 | 32·0 | 13 | 6 | 46·1 |
| III | 13 | 1 | 7·9 | 15 | 5 | 33·3 |
| IV | 4 | 0 | 0·0 | 7 | 2 | 28·6 |
| Total | 52 | 14 | 26·9 | 43 | 19 | 44·2 |

in Tables VI–VIII respectively. The 5 year survival when considered in relation to both Broder's grade and extent, and both histological cell type and extent, is shown in Tables IX and X respectively.

Of the 193 cases with DNA values of less than 155, the 5 year survival was 38·6%. In contrast, the 5 year survival was 56·8% among 169 cases in which the DNA values exceeded 155. Tables XI and XII cite the 5 year survival in relation to cell type, extent of the tumour and DNA values of less than 155 and DNA values that exceeded 155. Of the 10 small cell carcinomata, 7 had DNA values of less than 155 and none survived 5 years whereas 1 of the 3 patients with DNA values exceeding 155 survived more than 5 years.

DISCUSSION

A subclassification of the morphological variants of a malignant tumour should serve 3 important functions. First, it must permit universal recognition and classification according to morphology for the purposes of uniform reporting and recording. Secondly, on the basis of the morphological classification it must provide information relating to the biological behaviour. As most invasive carcinomata of the uterine cervix are treated by ionizing radiation, a second function of a morphological classification of malignant tumours occurring at this site might relate to radiosensitivity or radiocurability. The morphological variants may also relate to amenability to surgical intervention or a combination of surgery and radiotherapy. Thirdly, based on current concepts, the morphological variants so classified will provide information relating to the carcinogenesis of each morphological type.

Invasive squamous carcinomata of the uterine cervix have been grouped in various ways in order to gain information about their relative malignancy. In 1890, von Hansemann noted that tumours composed of poorly differentiated cells were in general more malignant than those composed of better differentiated cells. Subsequent grading or classification systems have been based on this observation. Broders (1926) was the first to utilize this principle in grading epidermoid cancers as to their relative content of differentiated cells. Those neoplasms containing from 75–100% differentiated cells were designated as Grade I while the least differentiated, containing up to 25% differentiated cells, were placed in grade IV. In this classification, keratinization was used as the index of differentiation. Similarly, Warren (1931) also graded tumours of the uterine cervix as to their relative content of differentiated cells, utilizing a somewhat more detailed scheme. As in Broders' classification, considerable importance was attached to keratinization as an index of differentiation.

Both Broders' and Warren's grading systems have proved useful in fulfilling the function of providing a uniform morphological classification. However, there is reason to believe that these grading systems are not reliable in predicting the biological behaviour or radiocurability of cervical cancer. In a series of 740 patients (Reagan, 1962) with invasive cancer of the uterine cervix treated before 1940 and classified histologically according to the method described by Warren, it was observed that there was no correlation between histological grade and survival. Of 207 patients with grade I cancers, 21·3% survived 5 years; of 309 patients with grade II cancer 26·9% survived 5 years, and of 224 patients with grade III cancer, 25·0% survived 5 years. The relatively low overall 5 year survival of 24·9% in this series was in large part due to a predominance of advanced cancers. Using the same method of grading, Kistner and Hertig (1951) reported an overall 5 year survival of 50% in a series of invasive carcinomata which included fewer advanced cases than the study cited above. However, they also concluded that the salvage rates were about the same in the 3 categories. It would appear from the foregoing and in this study that when keratinization is used as an index of differentiation for the classification of invasive carcinoma of the uterine cervix, there is relatively little correlation between differentiation and biological behaviour and/or radiocurability. A similar conclusion was also reported by Sidhu, Koss and Barber (1970).

In 1923 Martzloff proposed a classification for carcinoma of the uterine cervix based on the resemblance of the neoplastic cells to normal cells composing the squamous mucosa of the uterine cervix. Martzloff's concept of a cellular classification for invasive cancers of the uterine cervix was developed further in the classification proposed by Wentz and Reagan (Reagan and Hicks, 1953; Wentz and Reagan, 1959), which included large cell non-keratinizing, keratinizing and small cell cancers.

The important features of this classification of carcinoma of the uterine cervix, when compared with other grading systems, are that it provides (1) a morphological classification which permits a correlation between tissue and cellular specimens (Reagan and Hicks, 1953; Patten, 1969); (2) information relating to the histogenesis of the tumours (Reagan and Wentz, 1967; Reagan, Ng and Wentz, 1969; Ng and Reagan, 1969); and (3) information relating to the radiosensitivity and/or radiocurability of the tumours and thus to the prognosis of the patients (Wentz, 1961; Wentz and Reagan, 1959; Bangle, Berger and Levin, 1963). Wentz and Reagan (1959) observed a 5 year survival of 78·6% in those cases classified as large cell non-keratinizing, 47·8% in keratinizing cancers and 20·0% in small cell cancers. Additional studies with comparable clinical staging showed that small cell cancers are less likely to survive compared with large cell non-keratinizing cancers. Those patients with keratinizing cancers had an intermediate survival rate. The findings in this and similar studies have supported the correlation of the cell types and 5 year survival. (Bangle *et al.*, 1963; Finck and Denk, 1970; Patten, 1969).

Morphologically, keratinizing squamous cell cancers according to Wentz and Reagan's classification would generally correspond to modified Broders' grade I cancer; large cell non-keratinizing carcinomata would correspond to either II or III and small cell cancers would usually be called Broders' grade III.

That the 5 year survival was highest in neoplasms with minimal spread and poor in advanced neoplasms was evident in this study. When the 5 year survival was considered in relation to both clinical stage of disease and cell type, a significant difference in survival according to cell type was apparent. The 5 year survival for both large cell and keratinizing cancer decreased proportionately with increasing extent of disease. The 5 year survival for stage I cancers of large cell and keratinizing cancers appeared comparable. However, there appeared to be a significant difference in 5 year survival between large cell cancer and keratinizing cancer in clinical stage II disease. This difference was less conspicuous for stage III and stage IV neoplasms. The limited number of small cell cancers precluded a meaningful comparison with extent and the other cell types. There was no meaningful correlation in the 5 year survival when the clinical stage of disease was considered in relation to Broders' grade.

Although the histological cell type and clinical stage of the neoplasm are important in the prognosis of squamous cell cancer of the uterine cervix, there is evidence to indicate that the DNA values of the neoplastic cells may further characterize the course of the neoplasm. The 5 year survival in women with squamous cell carcinoma in which the DNA value of neoplastic cells exceeded 155 was more favourable than those with DNA values of less than 155. This finding was similar to that previously reported by Atkin (1971). Furthermore in this series, the difference in DNA modal values in relation to 5 year survival was apparent for each histological cell type and clinical stage of the disease. The explanation for this difference is not clear. It is possible that cells from squamous cell cancers showing high DNA values reflected a high turnover or mitotic rate at the time of detection and such tumour cells might be more susceptible to irradiation therapy. However, since a series of squamous cell carcinomata of the cervix treated by surgery alone had a better prognosis with fewer lymph node metastases when the nuclear size was large (the latter presumably indicating a high modal DNA value), the important factor relating DNA value to prognosis may be the earlier metastatic spread of those tumours with low values (Atkin and Richards, 1962). Thus, based on the evidence of this study, the clinical stage of the disease, histological cell type and the DNA values of neoplastic cells appeared to be important factors in evaluating the biological behaviour of squamous cell carcinoma of the uterine cervix.

We thank Dr M. H. Bennett for providing us with the histological sections of these cases. One of us (N.B.A.) is in receipt of a personal grant from the Cancer Research Campaign.

### REFERENCES

ATKIN, N. B. (1971) Cytogenetic Factors Influencing the Prognosis of Uterine Carcinoma. In *Modern Radiotherapy: Gynaecological Cancer*, 2nd Edn. Ed. T. J. Deeley. London: Butterworth and Co. p. 138.

ATKIN, N. B. & RICHARDS, B. M. (1962) Clinical Significance of Ploidy in Carcinoma of Cervix: its Relation to Prognosis. *Br. med. J.*, ii, 1445.

ATKIN, N. B. & RICHARDS, B. M. (1956) Deoxyribonucleic Acid in Human Tumours as Measured by Microspectrophotometry of Feulgen Stain: a Comparison of Tumours Arising at Different Sites. *Br. J. Cancer*, **10**, 769.

ATKIN, N. B., MATTINSON, G. & BAKER, M. C. (1966) A Comparison of the DNA Content and Chromosome Number of Fifty Human Tumours. *Br. J. Cancer*, **20**, 87.

ATKIN, N. B., RICHARDS, B. M. & ROSS, A. J. (1959) The Deoxyribonucleic Acid Content of Carcinoma of the Uterus: an Assessment of its Possible Significance in Relation to Histopathology and Clinical Course, Based on Data from 165 Cases. *Br. J. Cancer*, **13**, 773.

BANGLE, R., BERGER, M. & LEVIN, M. (1963) Variations in the Morphogenesis of Squamous Carcinoma of the Cervix. *Cancer, N.Y.*, **16**, 1151.

BRODERS, A. C. (1926) Carcinoma: Grading and Practical Application. *Archs Path.*, **2**, 376.

FINCK, F. M. & DENK, M. (1970) Cervical Carcinoma: Relationship between Histology and Survival Following Radiation Therapy. *Obstet. Gynec. N.Y.*, **35**, 339.

KISTNER, R. W. & HERTIG, A. T. (1951) A Correlation of Histologic Grade, Clinical Stage, and Radiation Response in Carcinoma of the Uterine Cervix. *Am. J. Obstet. Gynec.*, **61**, 1293.

MARTZLOFF, K. H. (1923) Carcinoma of the Cervix Uteri: a Pathological and Clinical Study with Particular Reference to the Relative Malignancy of the Neoplastic Process as indicated by the Predominant Type of Cancer Cell. *Bull. Johns Hopkins Hosp.*, **34**, 141.

NG, A. B. P. & REAGAN, J. W. (1969) Microinvasive Carcinoma of the Uterine Cervix. *Am. J. clin. Path.*, **52**, 511.

PATTEN, S. F. JR (1969) *Diagnostic Cytology of the Uterine Cervix.* Baltimore: Williams and Wilkins Co. p. 155.

REAGAN, J. W. (1962) Recent Advances in the Use of Cytological Studies in the Diagnosis of Cancer of the Uterus. *Acad. med. New Jersey Bull.*, **8**, 210.

REAGAN, J. W. & HICKS, D. M. (1953) A Study of *in situ* and Squamous Cell Cancer of the Uterine Cervix. *Cancer, N.Y.*, **6**, 1200.

REAGAN, J. W. & WENTZ, W. B. (1967) Genesis of Carcinoma of the Uterine Cervix. *Clin. Obstet. Gynec.*, **10**, 883.

REAGAN, J. W., NG, A. B. P. & WENTZ, W. B. (1969) Concepts of Genesis and Development in Early Cervical Neoplasia. *Obstet. Gynec. Survey*, **24**, 860.

SIDHU, G. S., KOSS, L. G. & BARBER, H. R. K. (1970) Relation of Histologic Factors to the Response of Stage I Epidermoid Carcinoma of the Cervix to Surgical Treatment. *Obstet. Gynec., N.Y.*, **35**, 329.

VON HANSEMANN, D. (1890) Ueber asymmetrische Zelltheilung in Epithelkrebsen und deren biologische Bedeutung. *Virchows Arch. path. Anat. Physiol.*, **119**, 299.

WARREN, S. (1931) The Grading of Carcinoma of the Cervix Uteri as Checked at Autopsy. *Archs Path.*, **12**, 783.

WENTZ, W. B. (1961) Histologic Grade and Survival in Cervical Cancer. *Obstet. Gynec., N.Y.*, **18**, 412.

WENTZ, W. B. & LEWIS, G. C. JR (1965) Correlation of Histologic Morphology and Survival in Cervical Cancer following Radiation Therapy. *Obstet. Gynec. N.Y.*, **26**, 228.

WENTZ, W. B. & REAGAN, J. W. (1959) Survival in Cervical Cancer with Respect to Cell Type. *Cancer, N.Y.*, **12**, 384.